# Promoter toolset for CRE-mediated conditioned expression of genes during somatic gonad development in *Caenorhabditis elegans*

**DOI:** 10.17912/micropub.biology.002121

**Published:** 2026-05-11

**Authors:** David Borrego, Adrian Fragoso-Luna, José Pérez-Martín

**Affiliations:** 1 Biomedical Institute of Valencia (CSIC), Valencia, Spain; 2 Institute for Functional Biology and Genomics (CSIC), Salamanca, Spain

## Abstract

This study examines the specificity and temporal dynamics of Cre drivers in *Caenorhabditis elegans* somatic gonad development. We use regulatory regions from factors known to be expressed within the Distal Tip Cell (DTC) lineage: *hnd-1 p* and *ceh-22 p* drivers exhibit broad expression throughout the somatic gonad; *lin-32 p* expression is confined to Z1a/Z4p derivatives; and *hlh-12 p* specifically targets the DTC. Temporal profiling shows *hnd-1 p* activity from early L1, *ceh-22 p* from early L2, and *lin-32 p* and *hlh-12 p* activity emerging later in L2/L3. Collectively, these results enable the application of Cre-mediated drivers to conditional expression in specific somatic gonad cell types.

**Figure 1. Spatio-temporal expression patterns of various Cre drivers during distal tip cell (DTC) differentiation.** **(A)** Diagram illustrating the transgenes utilized in this study. The left scheme represents the Cre drivers, while the central and right schemes depict the two distinct Cre reporters employed. **(B)** Representative images of the central region of young adult worms expressing the indicated Cre drivers and an integrated Cre reporter system. mCherry fluorescence marks cells with Cre activity. Solid lines delineate the worm body, while a dotted white line outlines the gonad. Somatic gonad cell types are labeled (DTC: distal tip cell, Sp: spermatheca). A minimum of 10 worms was analyzed for each combination, consistently yielding the same pattern as observed in the representative image. Scale bar: 50 μm. **(C)** Images of the somatic gonad primordium (SGP) in worms expressing the indicated Cre drivers at various developmental stages following release of bleaching-synchronized L1 larvae onto bacteria-seeded NGM plates. The SGP is outlined by a dotted white line encompassing mCherry-fluorescent germline cells. GFP-fluorescent nuclei indicate Cre activity. A minimum of 10 worms was analyzed for each combination and timing, consistently yielding the same pattern as observed in the representative image. The schematic on the left summarizes GFP-positive nuclei (green dots) at 24 hours. Scale bar: 20 μm. **(D)** Summary diagram illustrating the temporal and spatial activity of each Cre driver, as determined from panels B and C.

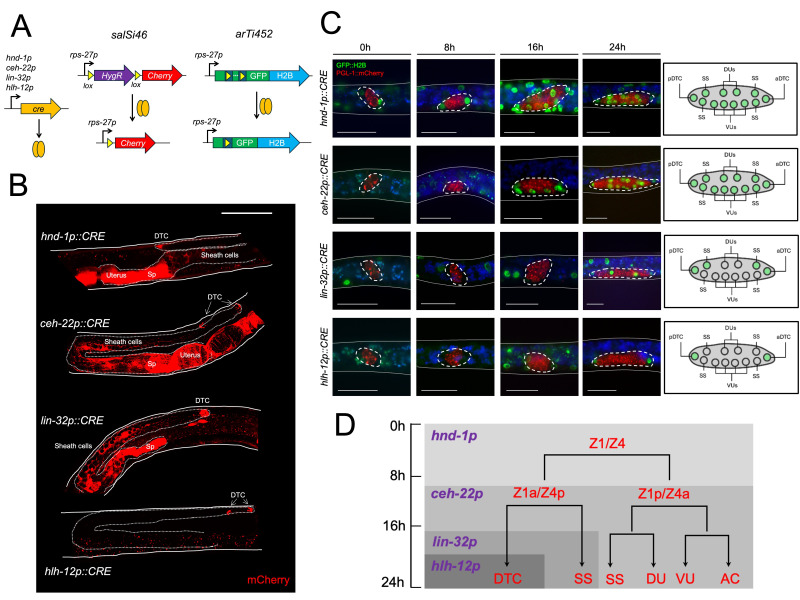



## Description

Research of animal development depends on the ability to observe and manipulate specific gene functions at defined times and locations. Therefore, tools that provide spatiotemporal control over gene expression are critical for studying the complex mechanisms underlying tissue and organ formation in multicellular organisms. Developing such tools requires the identification and characterization of tissue- and lineage-specific promoters that regulate gene transcription in targeted cell populations. Utilizing these promoters to drive the expression of site-specific recombinases, such as Cre or FLP, in combination with stop cassettes flanked by recombinase recognition sites and inserted into the gene of interest, represents a versatile approach for conditional gene expression (Hubbard, 2014; Shaffer and Greenwald, 2022). This strategy enables comprehensive genetic analysis of various biological processes and allows researchers to avoid embryonic lethality and pleiotropic effects often associated with conventional null mutations.

A significant advantage of using the nematode *Caenorhabditis elegans* as an experimental model is the extensive knowledge of its cell lineages and the regulatory factors that govern their temporal progression (Liu and Murray, 2023). This detailed understanding facilitates the identification of lineage- and tissue-specific promoters, which supports the development of tools for precise spatiotemporal control of gene expression. Furthermore, the invariant nature of the somatic cell lineage in *C. elegans* offers a unique opportunity to study cell-fate decisions and developmental plasticity at single-cell resolution.

Gonadogenesis in *C. elegans* provides a robust model for studying organogenesis, cell migration, and cell fate specification. The somatic reproductive system of the adult hermaphrodite originates from two somatic gonadal precursors (SGPs), Z1 and Z4 (Kimble and Hirsh, 1979). Together with the two primordial germ cells (PGCs), Z2 and Z3, they form the four-cell gonadal primordium present in the late embryo. Z1 and Z4 act as multipotent progenitors that generate all somatic cell types of the adult gonad, including the gonadal sheath, uterus, spermatheca, spermatheca-uterine valve, and distal tip cells (DTCs). During late embryogenesis, the SGPs migrate to associate with the PGCs, partially enveloping them. Following hatching at the L1 and L2 stages, successive asymmetric divisions of Z1 and Z4 and their descendants produce 12 cells that constitute the somatic gonadal primordium in the L2 stage. These 12 cells comprise two DTCs, which are essential for gonad elongation and germ-line patterning; nine blast cells that collectively generate all other adult somatic gonad cells; and the transient anchor cell (AC), responsible for patterning the vulval cells. In the L3 stage, further divisions of the somatic gonad blast cells produce 28 additional somatic cells, completing the organ's architecture. The precise and invariant lineage of these cells has been extensively mapped, providing a high-resolution framework for analyzing the roles of cell-cell interactions and intrinsic genetic programs in developmental progression.

We are interested in the genesis of the distal tip cell (DTC), with particular emphasis on the putative role of cell cycle regulators in its differentiation. DTCs are generated through two successive asymmetric divisions of Z1 and Z4, a process regulated by a Wnt signal gradient (Sawa and Korswagen, 2013). In contrast to their sister spermatheca/sheath (SS) blast cells, DTCs do not undergo further divisions. We believe that cell cycle regulators involved in cell cycle exit may play key roles in DTC determination. To help investigate this hypothesis, we have constructed a toolset of promoters to drive Cre recombinase at each cell division within the DTC lineage together with a fluorescent reporter gene to detect Cre-mediated recombination ( Figure 1A ). Specifically, 5' regulatory regions from genes encoding transcriptional regulators present in each of the DTC precursor cells were selected: *hnd-1* (Z1/Z4), *ceh-22* (Z1a/Z4p), and *lin-32* and *hlh-12* (Z1aa/Z4pp) (Mathies et al., 2003; Lam et al., 2006; Sallee et al., 2017). The corresponding DNA regions were cloned upstream of a Cre recombinase gene optimized for *C. elegans* (Dickinson et al., 2015) in a Mos universal vector for integration by MosSCI at *oxTi444* III (Frøkjær-Jensen et al., 2014). In addition, a Cre-dependent reporter was constructed, comprising an mCherry gene regulated by the strong, ubiquitous *rps-27* promoter ( *rps-27 p* ) and an intervening stop cassette flanked by *lox* sites positioned between the *rps-27* promoter and the mCherry translation start site. Upon Cre activity, the intervening stop cassette is excised, which enables expression of the mCherry fluorophore ( Figure 1A ). The transgene was integrated using MosSCI at *oxTi177* IV.

The combination of the Cre driver and the fluorescent reporter enabled sustained and specific expression of mCherry in defined cell types within the adult somatic gonad. The expression pattern was determined by the specific Cre driver used. Both *hnd-1 p-* and *ceh-22 p-* driven Cre induced mCherry fluorescence throughout the somatic gonad. In contrast, *lin-32 p* -driven Cre restricted mCherry expression to cells derived from Z1a/Z4p, including the distal tip cell (DTC), sheath cells, and the spermatheca. Additionally, *hlh-12 p* -driven Cre limited fluorescent expression exclusively to the DTC ( Figure 1B ).

The timing of Cre expression under different drivers was also examined. To achieve this, Cre-driving transgenes were combined with a strain carrying a *pgl-1* mCherry-tagged allele, which marks germline cells, and a flexon-based reporter for Cre. The flexon consisted of an artificial exon containing stop codons in all three reading frames. This exon, flanked by *lox* sites, is included in an intron to be inserted into the target gene. Without Cre activity, the exon is retained in the mature mRNA, resulting in premature termination in all reading frames and triggering nonsense-mediated mRNA decay, thereby preventing protein translation. When Cre is present, the recombinase excises the exon, leaving the intron with a single *lox* site that is subsequently removed from the mature mRNA, thereby restoring gene expression (Shaffer and Greenwald, 2020). We have used a reporter consisting of a GFP-H2B fusion carrying a flexon and inserted at chromosome I ( *arTi452* , Wittes and Greenwald, 2024) ( Figure 1 A).

The somatic gonad primordium (SGP) was analyzed from bleaching-synchronized worms carrying the various Cre driver/reporter combinations at multiple time points after release onto bacterial-seeded NGM plates at 20ºC: 0 h (early L1), 8 h (middle L1), 16 h (early L2), and 24 h (L2/L3 transition) (Porta-de-la-Riva et al., 2012). SGP in worms with *hnd-1 p-* and *ceh-22 p-* drivers exhibited 12 GFP-fluorescent nuclei at 24 h, representing all descendants from Z1/Z4 at this stage. The *hnd-1 p* driver was active from early L1, whereas the first signal of *ceh-22 p* -driven Cre1 activity was detected at 16 h (early L2). At 24 h, *lin-32 p* -driven SGPs showed 4 GFP-positive nuclei corresponding to 2 DTCs and their SS (spermatheca/sheath) sisters, while the *hlh-12 p* -driver marked only 2 nuclei corresponding to the DTCs. In both cases, no GFP signal was detected at earlier time points ( Figure 1C ). These results are consistent with above observed pattern for mCherry expression in somatic gonad cell types, which differ according to the Cre driver employed ( Figure 1B ).

Collectively, these results support a proposed timeline for the use of Cre-mediated drivers to conditional gene expression along the DTC lineage ( Figure 1D ).

## Methods

**Plasmid Construction**

The plasmid pMOSII-CRE was constructed as a recipient vector for promoters from various genes. The pMOSII vector, a derivative of pCFJ350 (Frøkjær-Jensen et al., 2014) containing an alternative Multicloning Site (MCS) (Puerta et al., 2025), served as the backbone. A 1.46 Kbp fragment from pDD268 (Dickinson et al., 2015), which includes the Cre coding sequence and the *tbb-2* 3' UTR, was amplified by PCR using primers with *Asc* I and *Spe* I recognition sites at the 5' and 3' ends, respectively. The Cre-containing fragment was then cloned into the corresponding *Sfi* I/ *Asc* I sites of pMOSII.

The 5' regulatory regions from the different genes were amplified with specific primers carrying *Sfi* I and *Asc* I recognition sites at their 5' and 3' ends, respectively. The 5' regulatory regions started in the nucleotide before the ATG from each gene and encompassed upstream sequences up to 1633 nt ( *hnd-1 p* ), 1240 nt ( *ceh-22 p* , considering the first ATG from *ceh-22 b* ), 2016 nt ( *lin-32 p* ), and 988 nt ( *hlh-12 p* ). The corresponding DNAs were inserted between *Sfi* I/ *Asc* I sites in the pMOSII-CRE plasmid to result in the specific Cre driving vectors

The mCherry Cre-dependent reporter consisted of several elements: an 840 nt DNA fragment flanked by *Sfi* I/ *Asc* I recognition sites containing the *rps-27* promoter; a 1477 nt fragment flanked by *Asc* I and *loxP* sites, which includes the Hygromycin resistance coding sequence followed by the *unc-54* 3' UTR; and a 1267 nt fragment flanked by *Asc* I/ *Spe* I sites containing the mCherry coding sequence and *tbb-2* 3' UTR. The fragments with the *rps-27* promoter and mCherry coding sequence were assembled into the MCS of pMOSII between the *Sfi* I and *Spe* I sites, after which the *Asc* I-flanked HygR intervening sequence was inserted.

**Strain construction**

Table 1 describes the strains used in this study. Transgenes were generated using MosSCI by injecting the appropriate donor plasmids (50 ng/μl) together with a mix carrying the plasmid-encoding mosase (pCFJ1532, 10 ng/μl) and plasmids encoding co-injection markers (pCFJ104, 10 ng/μl; pGH8, 10 ng/μl; pCFJ90, 2.5 ng/μl; pMA122, 10 ng/μl) into EG8080 ( *oxTi444* III) and EG8081 ( *oxTi177* IV) strains (Frøkjær-Jensen et al., 2014). Transgenic worms were analyzed by diagnostic PCR using the primer pairs UNIMOS-1/UNIMOS-2 for the left flanking border and UNIMOS-3/UNIMOS-4 for the right flanking border (see Table 2). Additional diagnostic PCR was performed to confirm the presence of specific transgenes, as indicated in Table 2. The resulting worms were backcrossed twice and then crossed to generate the desired gene combinations.

The GFP flexon allele ( *arTi452* , I) and the mCherry-tagged *pgl-1* allele ( *cer70 ,* IV) were sourced from the GS9847 and CER414 strains, respectively (Wittes and Greenwald, 2024; Brena et al., 2020).

All strains will be deposited at the CGC or available upon request.

**Table 1**

**Table:** 

STRAIN	GENOTYPE
JPM666	*salSi86* [ *hnd-1 p::cre:: tbb-2 3'UTR* + *unc-119* (+)] III
JPM297	*salSi59* [ *ceh-22 p::cre:: tbb-2 3'UTR* + *unc-119* (+)] III
JPM206	*salSi49* [ *lin-32 p::cre:: tbb-2 3'UTR* + *unc-119* (+)] III
JPM205	*salSi48* [ *hlh-12 p::cre:: tbb-2 3'UTR* + *unc-119* (+)] III
JPM182	*salSi46* [ *rps27p::lox::hygro::lox::cherry:: tbb-2 3'UTR* + *unc-119* (+)] IV
JPM229	*salSi86* III *, salSi46* IV
JPM189	*salSi59* III *, salSi46* IV
JPM207	*salSi49* III *, salSi46* IV
JPM208	*salSi48* III *, salSi46* IV
JPM560	*arTi452* [ *rps-27 p::GFP (flexon):: H2B:: unc-54 3'UTR* ]I, *salSi86* III *, pgl-1 ( cer70* [ *pgl-1 ::mCherry* ]) IV
JPM524	*arTi452* I, *salSi59* III *, pgl-1* ( *cer70* ) IV
JPM559	*arTi452* I, *salSi49* III *, pgl-1* ( *cer70* ) IV
JPM604	*arTi452* I, *salSi48* III *, pgl-1* ( *cer70* ) IV

**Table 2**

**Table:** 

Allele	Primers	Size of PCR fragments (Kpb)
Left flanking border for Mos Universal insertions	UNIMOS-1: 5'GAGAATGGCATTGATATTAAGTGTATCTGC3' UNIMOS-2 5'AAGGACTTGGATAAATTGGCTCAAGCCTGC3'	WT: none Insert: 1.4
Right flanking border for Mos Universal insertions	UNIMOS-3 5'CACTAGTGAGTCGTATTACGTAGCTTGGCG3' UNIMOS-4 5'CGGGAGGCGAACCTAACTGTAAAAGTCCAC3'	WT: none Insert: 1.57
ChrIII Mos insertion site	ChrIIIDIR: 5'CATCGCTCGAAAGAAGAAGCCGCCCCGTCA3' ChrIIIREV: 5'TCAAGTCTGTTATTCCGAATGTCATGTCAC3'	WT: 0.7 Insert: none
*salSi86*	Phnd1DIR: 5'CTACACAGATCCTCCCACCATCGTGAACCT3' CreREV: 5'CACGAACGTCTTCTGGCTCGGCTGGGAACC3'	WT: none Insert: 0.37
*salSi59*	Pceh22DIR: 5'AAGTTATTTGAAACTCTTGAATCGCCGCTT3' CRE REV: 5'CACGAACGTCTTCTGGCTCGGCTGGGAACC3'	WT: none Insert: 0.33
*salSi49*	Plin32DIR: 5'AATGGCAGATAATTAATCACCTTGCCTCCT3' CreREV: 5'CACGAACGTCTTCTGGCTCGGCTGGGAACC3'	WT: none Insert: 0.39
*salSi48*	Phlh12DIR: 5'AGAAGGGTAACATGTGTGAAGCAGGTGGCT3' CreREV: 5'CACGAACGTCTTCTGGCTCGGCTGGGAACC3'	WT: none Insert: 0.39
ChrIV Mos insertion site	ChrIV DIR: 5'CAACTACCTCCGATCTCAAATTGCTCTAGG3' ChrIV REV: 5'AATAAACATGTAAACTCGAAACACTTGAGC3'	WT: 0.84 Insert: none
*salSi46*	Prps27DIR: 5'GCCTAGATTTGTGATTCTACCAAGTGGAAT3' HYGREV: 5'GCGGCCGATGCAAAGTGCCGATAAACATAA3'	WT: none Insert: 0.69

**Image acquisition**

For live imaging, worms were mounted between slide and coverslip on 2% agarose pads in M9 amended with 10 mM sodium azide as immobilization agent. Images were obtained using a Nikon Eclipse 90i fluorescence microscope with a Hamamatsu Orca-ER camera driven by Metamorph (Universal Imaging, Downingtown, PA). The objectives used were Plan Fluor 40x/0.75 ( Figure 1B ) and Plan Apo 60x/1.40 oil ( Figure 1C ). Images were further processed with ImageJ vs 1.53k software.
